# Integrative analysis of bulk and single-cell gene expression profiles to identify tumor-associated macrophage-derived CCL18 as a therapeutic target of esophageal squamous cell carcinoma

**DOI:** 10.1186/s13046-023-02612-5

**Published:** 2023-02-27

**Authors:** Xinghua Sui, Chunxia Chen, Xiuman Zhou, Xueyan Wen, Chao Shi, Guanyu Chen, Juan Liu, Zhuoying He, Yongjie Yao, Yin Li, Yanfeng Gao

**Affiliations:** 1 School of Pharmaceutical Sciences (Shenzhen), Shenzhen Campus of Sun Yat-Sen University, Shenzhen, 518107 China; 2 School of Life Sciences, Zhengzhou University, Zhengzhou, 450001 China; 3 Department of Molecular Pathology, The Affiliated Cancer Hospital of Zhengzhou University/Henan Cancer Hospital, Zhengzhou, 450008 China; 4 Thoracic Surgery Department, National Cancer Center/National Clinical Research Center for Cancer/Cancer Hospital, Chinese Academy of Medical Sciences and Peking Union Medical College, Beijing, 100021 China

**Keywords:** Esophageal squamous cell carcinoma, Tumor microenvironment, Cell–cell interaction, Tumor associated macrophage, CCL18

## Abstract

**Background:**

Esophageal squamous cell carcinoma (ESCC) is a common gastrointestinal malignancy with poor patient prognosis. Current treatment for ESCC, including immunotherapy, is only beneficial for a small subset of patients. Better characterization of the tumor microenvironment (TME) and the development of novel therapeutic targets are urgently needed.

**Methods:**

In the present study, we hypothesized that integration of single-cell transcriptomic sequencing and large microarray sequencing of ESCC biopsies would reveal the key cell subtypes and therapeutic targets that determine the prognostic and tumorigenesis of ESCC. We characterized the gene expression profiles, gene sets enrichment, and the TME landscape of a microarray cohort including 84 ESCC tumors and their paired peritumor samples. We integrated single-cell transcriptomic sequencing and bulk microarray sequencing of ESCC to reveal key cell subtypes and druggable targets that determine the prognostic and tumorigenesis of ESCC. We then designed and screened a blocking peptide targeting Chemokine C–C motif ligand 18 (CCL18) derived from tumor associated macrophages and validated its potency by MTT assay. The antitumor activity of CCL18 blocking peptide was validated in vivo by using 4-nitroquinoline-1-oxide (4-NQO) induced spontaneous ESCC mouse model.

**Results:**

Comparative gene expression and cell–cell interaction analyses revealed dysregulated chemokine and cytokine pathways during ESCC carcinogenesis. TME deconvolution and cell interaction analyses allow us to identify the chemokine CCL18 secreted by tumor associated macrophages could promote tumor cell proliferation via JAK2/STAT3 signaling pathway and lead to poor prognosis of ESCC. The peptide Pep3 could inhibit the proliferation of EC-109 cells promoted by CCL18 and significantly restrain the tumor progression in 4-NQO-induced spontaneous ESCC mouse model.

**Conclusions:**

For the first time, we discovered and validated that CCL18 blockade could significantly prevent ESCC progression. Our study revealed the comprehensive cell–cell interaction network in the TME of ESCC and provided novel therapeutic targets and strategies to ESCC treatment.

**Supplementary Information:**

The online version contains supplementary material available at 10.1186/s13046-023-02612-5.

## Background

Esophageal cancer ranked the sixth among malignant tumors worldwide. Over 90% of the tumor cases are esophageal squamous cell carcinoma (ESCC) [1, 2]. Despite recent advances in the personalized therapies, molecular subtyping and development of targeted drugs such as epithermal growth factor receptor (EGFR) inhibitor Gefitinib and anti-programed cell death protein-1 (anti-PD-1) pembrolizumab [3, 4], the prognosis of ESCC remains relatively poor with a 5-year survival rate of below 30% [5]. Therefore, it is urgent and important to identify early diagnosis biomarkers and therapeutic targets of ESCC.

So far, large-scale whole-exome sequencing of ESCC have unveiled several high frequency of mutations (e.g., TP53 and CDKN2A) and mutational processes (e.g., cell cycle and PI3K-AKT pathways) [6, 7]. However, such genomic studies have limitation to illustrate the evolution of TME [8]. Recent studies found somatic mutant clones colonize more than half of the normal esophageal epithelium during aging [9], which may explain the clinical limitations of genomic studies on tumor cells [10, 11].

Tumor microenvironment consists various immune cells and stromal cells, which play crucial roles in cancer progression and therapeutic responses [12, 13]. It has been shown that the infiltration of immune cells is directly related to the prognosis of cancer patients [14, 15]. For example, tumor associated macrophages (TAMs) can promote angiogenesis, progression and metastasis of breast cancer and hepatic carcinoma [16–18], by releasing a variety of chemokines and inflammatory factors. Myeloid derived suppressor cells (MDSCs) can promote tumor invasion and metastasis [19, 20] through inducing immunosuppressive regulatory T cells (Tregs) [21], directly inhibiting T cell activation [22] and the function of natural killer cells (NKs) [23]. Hence, a better understanding of immune cell biology within the ESCC microenvironment will help to elucidate the potential mechanism of tumorigenesis and responsiveness to immunotherapies.

The conventional methods to characterize the cell content of TME are fluorescence-activated cell sorting (FACS) and immunohistochemistry (IHC) staining. However, FACS requires a large amount of tumor biopsies and IHC can only reflect limited markers of a single tissue slice. To address these drawbacks, recent studies have utilized single-cell RNA sequencing (scRNA-seq) to dissect heterogeneous tumors and decipher the interaction of ESCC TME components [24, 25]. Despite the power of this technology, scRNA-seq is currently impractical on large sample cohorts due to the requirement of a large sample number and high cost. As most clinical specimens cannot be dissociated into intact single-cell suspensions, computational methods (such as CIBERSORTx and xCell) were developed to enable characterization of large-scale tissue using cell signatures derived from single-cell reference profiles [26–28].

**Fig. 1:**
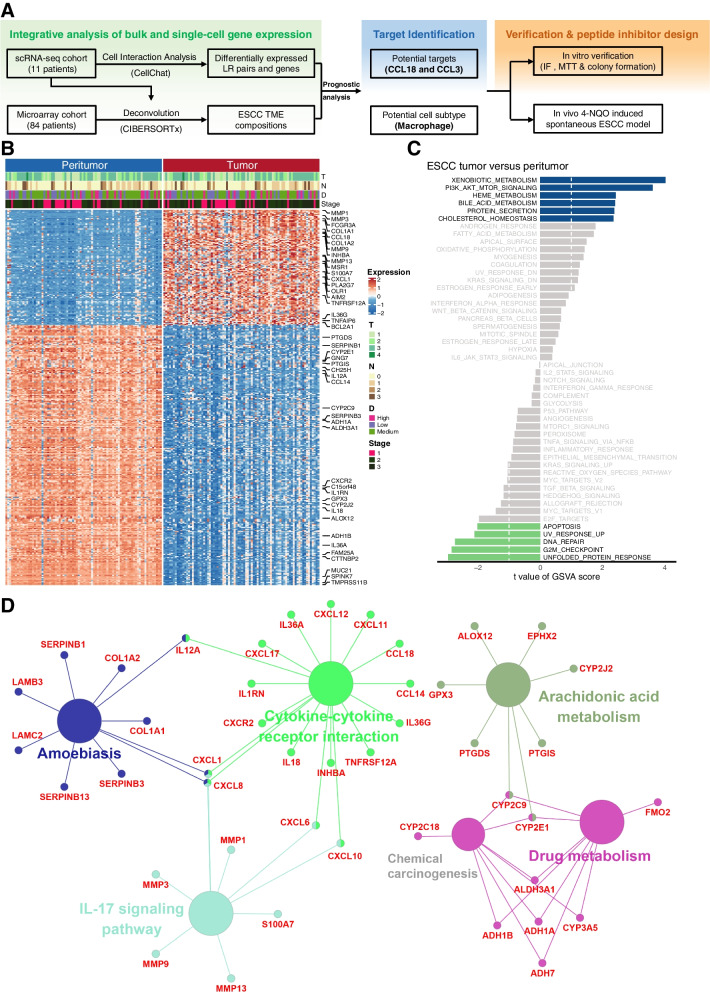
Design overview and molecular characteristics of the ESCC microarray cohort. (**A**) Schematic diagram of the study design. (**B**) 351 differentially expressed genes with log fold change > 2 and *P* < 0.01 between paired peri- and tumor samples in ESCC were shown as heatmap with differential degree (D), tumor (T), nodal (N) and stages information. (**C**) Differences in hallmark pathway activities scored by GSVA between paired peri- and tumor samples in ESCC. Shown are t values from a linear model, corrected for patient of origin. (**D**) KEGG Pathway enrichment analysis result of the differentially expressed genes in ESCC

In the present study, we hypothesized that the integration of single-cell transcriptomic sequencing and large microarray sequencing of ESCC biopsies would reveal the key cell subtypes and therapeutic targets that determine the prognosis and tumorigenesis of ESCC. We characterized the gene expression profiles, gene sets enrichment, and the TME landscape of a microarray cohort including 84 ESCC tumors and their paired peritumor samples. We then utilized CIBERSORTx method with publicly available ESCC scRNA-seq dataset to deconvolute the TME of the ESCC microarray cohort and examine the prognostic value of TME components. We identified dysregulated chemokine and cytokine pathways during ESCC tumorigenesis with comparative analysis of molecular signaling combing differential expressed gene analysis and cell–cell interaction analysis. The comprehensive TME and immune landscape analyses allow us to identify the chemokine CCL18 (Chemokine C–C motif ligand 18) derived from TAMs, which could promote tumor cell proliferation and lead to poor prognosis of ESCC. We then designed and screened a CCL18 blocking peptide and validated its potency by MTT assay. The antitumor activity of CCL18 blocking peptide was validated in vivo by using 4-nitroquinoline-1-oxide (4-NQO)-induced spontaneous ESCC mouse model (Fig. 1A). For the first time, our results demonstrated that the CCL18 blockade could significantly prevent ESCC progression, indicating that inhibition of CCL18 could be a novel strategy for the treatment of ESCC.

## Materials and methods

### ESCC microarray cohort and scRNA-seq data analysis

Paired tumor and peritumor tissues were collected from 84 ESCC patients. Peritumor samples were collected 2–5 cm from the edge of tumor tissue. All patients were surgically proven primary ESCC, and the follow-up was performed by the Henan Cancer Hospital. The detailed clinical and pathological information for the patients were listed in Table 1. Samples were obtained with informed consent, and the study was approved by the Ethics Committee of Henan Cancer Hospital.

**Table 1:** Clinical and pathological features of patients in ESCC microarray cohort and correlations of CCL18 expression levels

Clinical features	N	CCL18 expression level		*P* value^*^
		**Low N (%)**	**High N (%)**	
Tumor location ^a^				
Upper	22	15 (17.6)	7 (8.3)	0.435
Middle	39	20 (23.8)	19 (22.6)	
Lower	22	13 (15.5)	9 (10.7)	
Tumor differentiation				
Well	25	14 (16.7)	11 (13.1)	0.569
Moderate	43	23 (27.4)	20 (23.8)	
Poor	16	11 (13.1)	5 (6.0)	
Pathological stage				
IA-IB	26	20 (23.8)	6 (7.1)	**0.043**
IIA-IIB	29	15 (17.6)	14 (16.7)	
IIIA-IIIC	29	13 (15.5)	16 (19.0)	
Tumor stage				
T1-T2	39	31 (36.9)	8 (9.5)	**0.0001**
T3-T4	45	17 (20.2)	28 (33.3)	
Nodal stage				
N = 0	48	29 (34.5)	19 (22.6)	0.484
N > 0	36	19 (22.6)	17 (20.2)	

^a^ exclude one patient whose tumor locate in the neck

^*^χ2 test

Total RNA of collected frozen samples was extracted and the gene expression profiling was performed using the Affymetrix microarray platform (PrimeView Human Gene Expression Array). For data processing, raw data were normalized using the Robust Multi-array Average procedure in the R package oligo. We implemented Bayes statistics using empirical Bayes (eBayes) in the limma package to identify expressed genes between paired tumor and peritumor samples with significant differences.

Single cell RNA-seq data of ESCC with 41,237 cells from PRJNA777911 [29] were selected for the analysis of cell–cell communication and to derive the deconvolution marker gene reference data source. Specifically, 19,882 ESCC primary tumor and 21,355 matched adjacent nonmalignant esophageal cells from 11 treatment naive ESCC patients (10 × Genomics Chromium, 3' assay) were reanalyzed in this study after removing potential doublets and cells with high mitochondrial content. Cell clusters were annotated by the references of the experiment metadata as T cells, B cells, epithelial/tumor cells, macrophages, monocytes, fibroblasts, endothelial cells, dendritic cells (DC), mast cells, and smooth muscle cells. We annotated and clustered diverse cell types of each cell following the original publication. Another scRNA-seq dataset (CRA002118) of 4-nitroquinoline 1-oxide (4NQO) induced mouse ESCC model [30] was also processed to investigate cell-type specific gene expression and cell–cell communication patterns. We selected 19,829 cells from 3 treatment naive mice, 3 mice with carcinoma in situ (CIS) and 3 mice with invasive carcinoma (ICA). Cell clusters were annotated according to the original publication as T cells, B cells, epithelial cells, fibroblasts, myocytes, myeloid and endothelial cells. The Uniform Manifold Approximation and Projection (UMAP) dimensionality reduction was then performed to visualize the result of clustering and the differentially expressed genes for all clusters were determined using R package Seurat.

### Data analysis and statistical methods

In brief, mRNA expression data in 84 paired tumor and peritumor samples were extracted by quantile normalization and were log2 scaled transformed. For genes with more than one probe, mean expression was calculated. Differentially expressed genes (DEGs) were characterized by log2 fold change of greater than 2, and an eBayes test *P* value was less than 0.01 using R package limma.

Student’s t-test, analysis of variance, and the Kruskal–Wallis test were utilized to compare continuous variables and ordered categorical variables. All tests were two sided, and a *P* value of less than 0.05 was considered as statistical significance unless stated otherwise. A Cox proportional hazard regression model adjusting or not adjusting for available prognostic clinical covariates was performed to calculate hazard ratios (HRs) and 95% confidence intervals. The *P* values were adjusted to false discovery rate (FDR) using the Benjamini–Hochberg method in multiple comparisons. The survival curves were compared using Kaplan–Meier method and log rank test. All data analyses and visualization were performed using R (version 4.0.1).

### Pathway enrichment analysis

Gene set enrichment analysis (GSEA) and Kyoto Encyclopedia of Genes and Genomes (KEGG) Gene Ontology (GO) annotation were performed using the R package clusterProfiler with the DEGs identified previously. Hallmark gene sets and canonical KEGG pathways gene sets were retrieved from the Molecular Signatures Database (v7.4). The enrichment scores of molecular pathways and gene expression signatures were evaluated using the single-sample gene set enrichment analysis (R package GSVA). KEGG Pathway enrichment analysis result was visualized using ClueGO (Version 2.5.6) within Cytoscape (Version 3.7.2).

### Deconvolution of ESCC microarray data

We used CIBERTSORTx [28] to infer cell type abundances from the ESCC microarray cohort. Briefly, we at first generated a CIBERSORTx signature matrix using cell types from the ESCC scRNA-seq dataset PRJNA799111 with 41,237 cells by applying the Create Signature Matrix module (https://cibersortx.stanford.edu/runcibersortx.php) with the log-normalized expression matrix of 10 major cell populations as a reference matrix with default parameters. Using the resulting signature matrix, CIBERSORTx deconvolution was performed on the ESCC microarray cohort with 1000 permutations and S-mode batch correction.

### Cell–cell interactions analysis

We used CellChat [31] to infer cell–cell communication by integrating ESCC scRNA-seq data with prior ligand-receptor interaction database CellChatDB and Omnipath [32]. CellChat at first identifies differentially over-expressed ligands and receptors for each cell group. Then quantifies the communication probability between two interacting cell groups based on the average expression values of a ligand by one cell group and that of a receptor by another cell group, as well as their cofactors. The significance of interactions is calculated using permutation tests. CellChat outputs an intercellular communication network for each ligand-receptor pair, with the calculated communication probabilities as edge weights representing the interaction strength.

The function computeCommunProbPathway inferred the cell–cell communication at a signaling pathway level. We used the netVisual_circle function to show the strength or weakness of cell–cell communication networks from the target cell cluster to different cell clusters in ESCC TME. The netVisual_bubble function shows the bubble plots of significant ligand-receptor interactions between the target cell cluster and other TME clusters.

To identify statistically significant upregulated and downregulated ligand-receptor pairs between tumor and peritumor sites, we combined cell–cell communication analysis with differential gene expression analysis. Specifically, for each cell group, we performed Wilcoxon rank-sum test for gene expression of cells in tumor samples *vs*. cells in peritumor. Signaling molecules are considered as upregulated in the second condition if (i) the *p* values are less than 0.05, (ii) the log fold-changes are higher than 0.1, and (iii) the percentage of cells with expression in the second condition is higher than 25%. Signaling molecules are considered as downregulated in the tumor samples if they are upregulated in the peritumor samples. The ligand-receptor pairs are upregulated or downregulated if both ligands and receptors are upregulated or downregulated.

### Cell lines

ESCC cell lines (KYSE-70, KYSE-140, KYSE-150, KYSE-450, EC-1, EC-109, EC-9706) and HET-1A, a normal human squamous esophageal epithelial cell line, were cultured in Roswell Park Memorial Institute (RPMI)-1640 (GIBCO, Grand Island, USA) with 10% fetal bovine serum (FBS) (BI, USA) and commercial antibiotic cocktail of penicillin and streptomycin (Procell, China) at 37 °C with 5% CO_2_ under fully humidified conditions.

### The induction of macrophages

Peripheral blood mononuclear cells (PBMCs) were obtained from healthy donors and isolated with Ficoll density gradient centrifugation. Cells were differentiated into monocyte derived macrophage (MDM) in vitro in the presence of 50 ng/mL M-CSF (Peprotech, USA) for 6 days. The MDMs were then cultured in medium containing 10 ng/mL M-CSF and polarized for 24 h by adding 20 ng/mL IFN-γ and 20 ng/mL LPS (M1) or 20 ng/mL IL-4 and 20 ng/mL IL-13 (M2). Cells were stained with the following antibodies, anti-human CD14 eVolve-605 (61D3, eBioscience), anti-human CD163 PE (eBiGHI/61, eBioscience), anti-human CD80 eFlour710 (2D10.4, eBioscience), and anti-human CD206 (MMR) FITC (15–2, Biolegend). The expression of cell surface molecules was analyzed by a BD FACS Celesta flow cytometer.

For the induction of murine macrophages BMDM, the bone marrow cells were isolated from the C57BL/6 J mice and cultured in the medium with 20 ng/mL of GM-CSF (Peprotech, USA) for 6 days, and the medium was refreshed every three days. The BMDMs were then cultured in medium containing 20 ng/mL GM-CSF for 24 h and further polarized by adding 20 ng/mL IFN-γ and 100 ng/mL LPS for M1 macrophages or 20 ng/mL IL-4 and 20 ng/mL IL-13 for M2 macrophages. The expression of mouse CCL3 in mRNA and protein levels were detected by qRT-PCR and ELISA, respectively. The primers 5’-TGACCTGGAACTGAATGCCT-3’ and 5’- TCAAGCCCCTGCTCTACACG-3’ were used for mouse CCL3. The primers for mouse PITPNM3 were 5’-TGATTCGGTGGAGAGTTCAGA-3’ and 5’-CTGGCTCATTCCAATAAGGATGG-3’, and primers 5’-GCATCCACTGGTGCTGCC-3’ and 5’-TCATCATACTTGGCAGGTTTC-3’ were used for the reference gene mouse GAPDH.

### The construction of PITPNM3 knockdown cell lines

For the knockdown of human PITPNM3, the shRNA sequence was designed and synthesized by Sangon Biotech, and then cloned into the lentiviral vector pSicoR-GFP. The inserting sequence was formed with the forward primer: 5’- TGCTGAGGAATGTCACGGCTAATCTCGAGATTAGCCGTGACATTCCTCAGTTTTTTC -3’ and reverse primer: 5’-TCGAGAAAAAACTGAGGAATGTCACGGCTAATCTCGAGATTAGCCGTGACATTCCTCAGCA -3’. To produce the lentivirus with shRNA, HEK-293 T cells were transfected with the packing vector pMD2.G (#12259, Addgene), psPAX2 (#12260, Addgene) and pSicoR-GFP or pSicoR-GFP-shPITPNM3. The lentivirus was collected and used to infect the EC-109 cells.

### Cell cycle and apoptosis assay

EC-109, EC-109 Vector and EC-109 shPITPNM3 cells were seeded into 6-well plates at a density of 3×10^5^ cells/well with or without the presence of 40 ng/mL rCCL18. With a 72 h treatment, the cells were harvest and stained with PI (Multi Sciences, China, CCS012), or stained with the cell apoptosis kit (Biogems, China, 72700–80) with Annexin V-APC and 7-AAD, according to the manufacturer’s instructions. The cells were acquired and analyzed by a BD FACSCelesta flow cytometer.

### MTT assay

The proliferation of EC-109 cells effected by the knockdown of PITPNM3, recombinant human rhCCL18 (Peprotech, USA), or the peptides were determined by MTT assay. Briefly, cells were seeded into a 96-well plate at a density of 3,000 cells/well. After 24, 48, and 72 h, cell viability was detected using MTT reagent (Sigma, USA) dissolved in 5 mg/mL of PBS 7.2 and incubated at 37 °C for 4 h. Then 150 μL of DMSO replaced the incubation medium. MTT reduction was quantified by measuring the absorbance at 490 nm.

### Cell colony formation assay

EC-109, EC-109 Vector and EC-109 shPITPNM3 cells were seeded into 6-well plates at a density of 500 cells/well, the cell culture media RPMI-1640 were refreshed every 4 days. Over 12 days, colonies were fixed with 4% polyformaldehyde solution and stained with 0.2% crystal violet (Solarbio, China). The visible colonies of each well were recorded and counted.

### Transwell

The Transwell assay was performed using a 24-well plate. EC-109, EC-109 Vector or EC-109 shPITPNM3 cells were seeded at a density of 5 × 10^5^ cells/well into the upper chamber within 200 μL medium without FBS, and the lower chamber was filled with 600 μL of medium containing 10% FBS. After 48 h incubation, cells were fixed with 4% paraformaldehyde, stained with crystal violet, and photographed.

### Wound healing

EC-109, EC-109 Vector or EC-109 shPITPNM3 cells were seeded at a density of 5 × 10^5^ cells/well into a 24-well plate. The monolayer cell was wounded by sterile tips to allow the formation of a cell-free path, and the suspended cells were washed. The cells were photographed at 0 h and 48 h, and the scratch areas were calculated.

### Western blot

EC-109, EC-109 Vector and EC-109 shPITPNM3 cells were treated with or without 40 ng/mL rCCL18 in the presence or absence of STAT3 inhibitor S3I-201 (MCE, China). The total cell lysates were prepared using protein lysis buffer. Then protein was fractionated by 10% SDS-PAGE, transferred to polyvinylidene fluoride (PVDF) membrane (Merck Millipore, IPVH00010, USA), and then blocked with 5% defatted milk dissolved in PBS (pH7.2) containing 0.1% Tween 20 for 2 h at room temperature. The PVDF membranes were incubated with the following primary human antibodies at 4 °C overnight: JAK2 (Abcam, AB32101, UK), P-JAK2 (Abcam, AB108596, UK), STAT3 (CST, 4904S, USA), P-STAT3 (CST, 9145 T, USA). The reference antibody was β-actin (Servicebio, China). Thereafter, the PVDF membranes were incubated with secondary antibody (Sangon Biotech, D110011-0100, China), the blots were visualized using ECL system (Azure C600, USA).

### Immunofluorescence

The tumor tissues from the human esophageal and the whole mouse esophageal tissues were fixed with 4% paraformaldehyde phosphate solution and were embedded in paraffin. The sections were then incubated with monoclonal antibodies against CD68 (1:5000, ZM0060, Servicebio, China), CD206 (1:5000, GB11032, Servicebio, China), and CCL18 (1:500, MAB394, R&D, USA). For trial staining, the primary antibodies were incubated one by one with HRP conjugated secondary antibody (1:500, appropriately respond to primary antibody in species, Servicebio, China) and fluorescein, whereafter the sections were heated by a microwave using an EDTA antigen retrieval buffer (pH 8.0) to remove the combined antibodies. Nucleus shows blue by staining with DAPI (G1012, Servicebio, China). Signals were detected by an Ortho-Fluorescent Microscopy (NIKON ECLIPSE C1, Nikon, Japan) and Imaging system (NIKON DS-U3, Nikon, Japan).

### Analysis of the sequence and structure of CCL18 and CCL3

The protein sequences of human CCL18, human and mouse CCL3 were obtained and aligned by ClustalW. The structures of the above proteins were taken from the PDB database. The human CCL18 (PDB ID: 4mhe, chain A) [33], human CCL3 (PDB ID: 3fpu, chain B) [34], and mouse CCL3 (PDB ID: 4mhe, chain F) were used. The N-terminal structure of mouse CCL3 was completed with the loop modeler function of the software Molecular Operating Environment (MOE). The sequence alignment was revealed with the online tool ESPript3 with the alignment file and the hCCL18 (4mhe) as the template. The protein was structurally superposed using the sequence alignment.

### Peptide design and synthesis

Peptides were selected from the N and C terminal of the human CCL18 and CCL3 according to the sequence alignment and the superposed structure. The two Cysteine residues in the N terminal were replaced by Serine to avoid the formation of disulfide bond. The peptides were synthesized by Fmoc-based solid-phase peptide synthesis (SPPS) according to the standard protocol and purified by high performance liquid chromatography (HPLC).

### Spontaneous ESCC mouse model

Female C57BL/6 J mice of 6–7 weeks aged were purchased from the Vital River Laboratory Animal Technology Co. Ltd (Beijing, China). Mice were housed in SPF animal facility at room temperature with humidity of 40–60% and light and dark cycle for 12 h. Ethical consent and experimental procedures were approved by the Ethics Committee Zhengzhou University (ethical approval code: ZZUIRB2021-32).

For the induction of spontaneous ESCC, the mice were fed with drinking water containing 100 μg/mL 4-nitroquinoline-1-oxide (4-NQO) for 16 weeks. Thereafter, the mice were fed with sterilized pure water to allow the formation of ESCC. During the whole experiment, the activity status, food and water intake behaviors, body weight, and survival status of mice were observed and recorded every 2 days. The whole esophageal tissues of mice fed for 16, 28 and 32 weeks were performed the H&E staining for the detection of the ESCC induction process. Besides, the total RNA of the esophageal tissues of mice fed for 28 weeks were extracted and used to determine the expression of CCL3, CCR1 and CCR5. Also, the esophageal tissues were subjected to immunofluorescence staining for DAPI, CCL3 and the macrophages (F4/80). At week 31, the 4-NQO induced ESCC mice were randomly divided into two groups, and were intraperitoneally injected with 200 μL of normal saline or 10 mg/kg Pep3 daily for 8 days. At the end of treatment, the esophageal tissues of mice were harvested and photographed by an anatomical microscope, and the number and length diameter of tumor lesions were recorded.

The spleen of the ESCC tumor bearing mice treated with NS or Pep3 were grinded and filtered into a single cell suspension. For the detection of the CD8^+^ T cells, CD4^+^ T cells and macrophages, the splenocyte was stained with the following antibodies purchased from eBioscience, anti-CD45 (30-F11), anti-CD3 (17A2), anti-CD8 (53–6.7), anti-CD11b (M1/70), anti-CD11c (N418), anti-F4/80 (BM8) and anti-CD206 (MR6F3). The mouse esophageal tissues were subjected to H&E staining and immunofluorescence staining for DAPI, Ki67, F4/80, and immunohistochemistry staining for PITPNM3 (Novus Biologicals, Littleton, USA, NBP2-33,894) by Servicebio (Wuhan, China).

## Results

### Molecular characteristics and cellular ecosystem in ESCC

The mRNA expression profiles of 84 paired treatment naïve ESCC samples were determined by microarray analysis. There were 351 differentially expressed genes (DEGs) with log2 fold change greater than 2 as shown in heatmap (Fig. 1B) with differential level (D), tumor (T), nodal (N) and stages information. Enrichment analysis of hallmark gene sets suggested that the upregulated genes in ESCC tumor were enriched in multiple metabolic related pathways, such as xenobiotic, bile acid and heme metabolism pathways, as well as PI3K/Akt/mTOR pathways (Fig. 1C). On the other hand, down-regulated pathways included DNA repair, G2M checkpoint and apoptosis pathways. Enriched KEGG pathways indicated that there were considerable changes in the genes related to the IL-17 signaling and cytokine-cytokine receptor interaction pathways, including cytokines of IL-6, IL-36A and IL-36G, tissue-destructive matrix metalloproteinases of MMP3, MMP9 and MMP13, and chemokine signaling and cytokine-cytokine receptor interactions of CXCL8, CXCR2, CCL18, CCL20 (Fig. 1D).

**Fig. 2:**
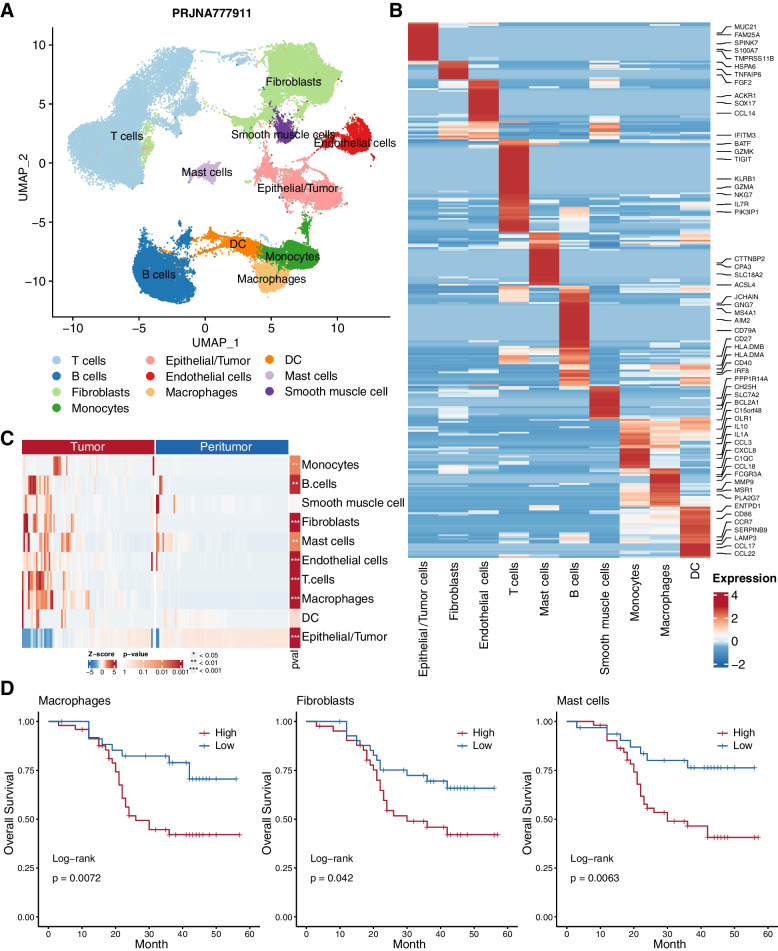
The immune landscape and prognostic significance of ESCC tumor microenvironment. (**A**) UMAP visualization of 41,237 cells from 22 peri- and tumor samples (PRJNA777911). (**B**) Heat map of signature matrix genes derived by CIBERSORTx from scRNA-seq data distinguishing 10 cell types. (**C**) Heatmap of the normalized absolute abundance for each cell type in the ESCC microarray cohort revealed distinct microenvironment compositions between peri- and tumor sites with t-test. (**D**) Kaplan–Meier curves of overall survival of ESCC patients stratified by the abundance of macrophages (left), fibroblasts (middle) and mast cells (right)

As ESCC ecosystem consists not only tumor cells, but stromal cells and immune cells [35], further investigation on cellular interactions between different cell populations within the ESCC microenvironment will help identify mechanisms underlying cellular responsiveness to immunotherapies. Previous single cell study [29] revealed heterogeneous ESCC microenvironment cell populations based on established markers, including epithelial cells (including both nonmalignant esophageal epithelial cells and tumor cells, termed Epithelial/Tumor), immune cells (monocytes, macrophages, dendritic, mast, T, and B cells), fibroblasts, smooth muscle cells, and endothelial cells (Fig. 2A).

To further explore the immune and prognostic landscape of ESCC microenvironment, we performed CIBERSORTx [28] to enumerate cellular fractions of bulk tissue by deconvolution of gene expression data using scRNA-seq-derived reference profiles. First we inferred cell-type expression signatures from annotated ESCC scRNA-seq dataset (PRJNA777911). There were 293 genes identified as cell type specific signatures (Fig. 2B), including 29 DEGs identified in the ESCC microarray cohort, such as MMP9, MMP12, CCL18, MSR1, CTTNBP2, and FCGR3A. Then we used the signature genes matrix to enumerate 10 cell types in the ESCC microarray. Monocytes, macrophages, fibroblasts, endothelial cells, B cells and T cells showed significant enrichment in the ESCC tumor sample with considerable inter-tumoral heterogeneity (Fig. 2C), reflecting a pro-inflammatory or immunosuppressive TME. We further investigated the prognostic association of 10 cell types in the ESCC microarray cohort. Although many studies have shown that the infiltration of different immune cells was related to the prognosis of cancer patients [21–23], our results showed that macrophages, fibroblasts, and mast cells were predictors of adverse outcomes of ESCC patients (Fig. 2D).

### Cell–cell communication analysis of ESCC TME

Our microarray and scRNA-seq analysis found the cytokine networks and extracellular matrix (ECM) played pivotal roles in ESCC TME. To gain further insight into local intercellular signaling in ESCC, we systematically searched for differentially expressed genes that coded for ligand and receptor/co-receptor pairs using the algorithm CellChat. CellChat infers cell–cell communications from scRNA-seq data by computing the expression of signaling ligands and receptor/co-receptors. We focused the study on cytokine- and chemokine-mediated interactions between epithelial/tumor and immune cells. To avoid missing any possible interactions, we at first updated ligand-receptor interaction database CellChatDB with Omnipathdb, leading to a comprehensive ligand–receptor database including 6260 unique connections. To investigate global differences in cell–cell communications between peritumor and ESCC tumors, we employed comparative CellChat (see Methods session) to study signaling interactions among all major cell types.

**Fig. 3:**
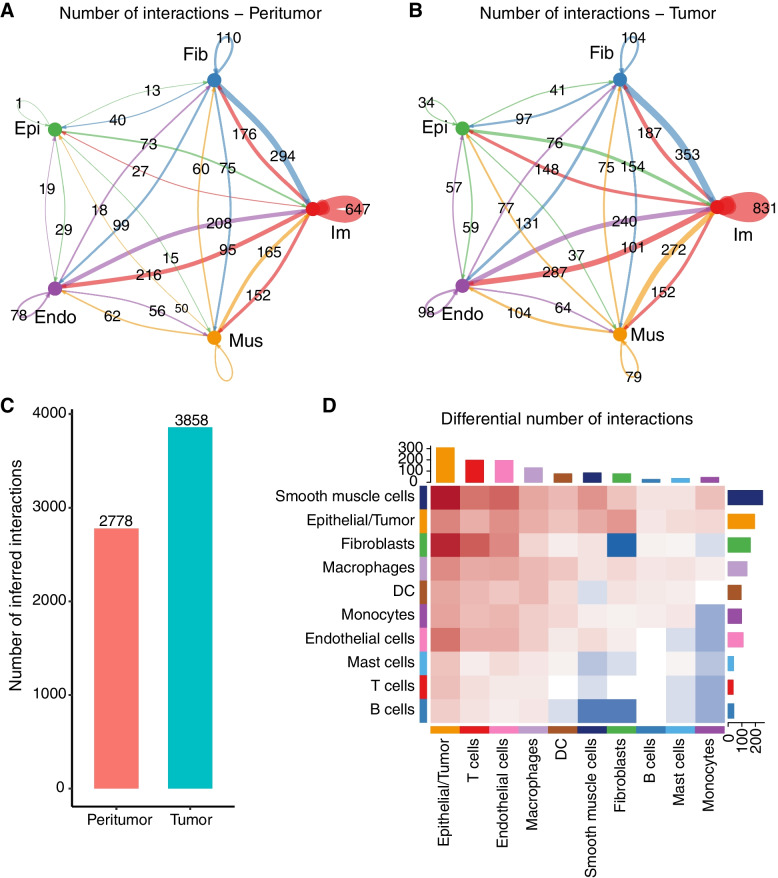
Cell–Cell communications in ESCC TME. Number of possible interactions between the five major cell types in peri- and tumor sites (**A** and **B**). (**C**) Total number of possible interactions. (**D**) Differential number of possible interactions between any two cell populations. Red (positive values) and blue (negative values) in the color bar indicate higher number of predicted interactions in peri- and tumor sites, respectively

We first examined the total number of possible cell–cell communications and the prominently affected cell types (Fig. 3A and B). There was a higher number of possible interactions in ESCC tumors than peritumors (Fig. 3C). Putative signaling within and between the epithelial/tumor and immune cells drastically increased in ESCC tumors (Figs. 3D, Fig. S2A and B). Moreover, signaling from macrophages to epithelial/tumor and T cells was also strengthened (Fig. S2C and D). These results showed that, in general, both autocrine and paracrine signaling probabilities among all three major cell types increased during ESCC tumorigenesis.

### Comparative analysis of cell communication

After gaining an understanding of the general cell–cell communications in ESCC tumors, we next investigated the molecular signaling mechanisms in deapth to identify potential pathways underlying the altered cellular compositions and communications in the ESCC ecosystem. We identified dysregulated ligand-receptor pairs in ESCC by combining differential expression analysis with cell–cell communication analysis. CellChat predicted significant upregulation of 24 ligand-receptor pairs from 11 signaling pathways in ESCC tumor tissues compared with peritumor tissues.

**Fig. 4:**
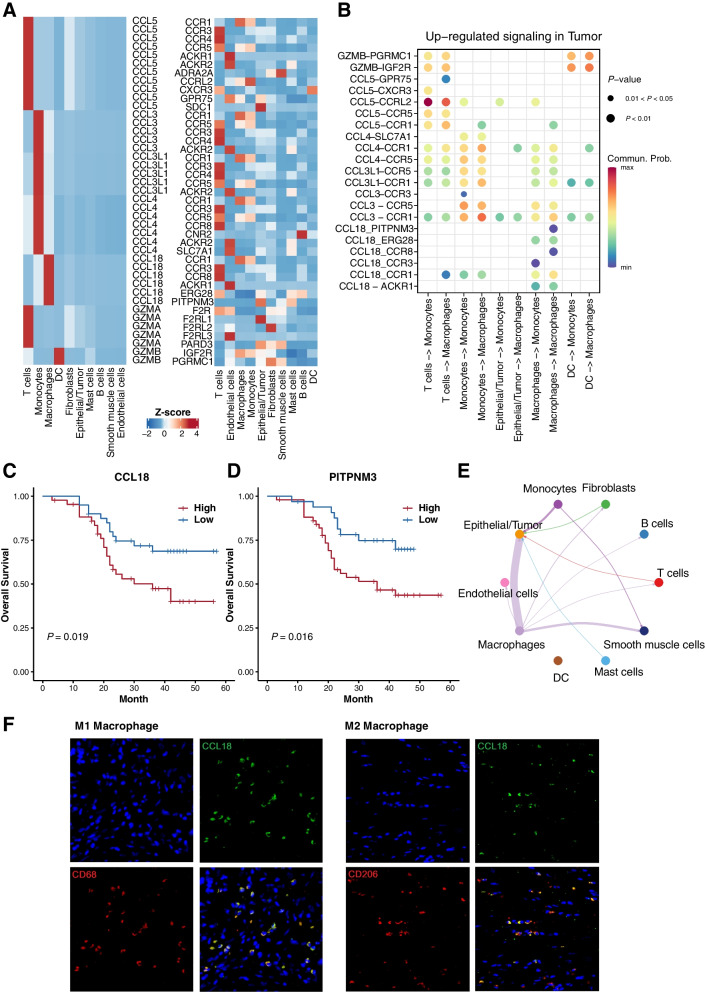
Comparative analysis of cell communication identified differentially expressed ligand-receptor pairs in ESCC. (**A**) Heatmap showing the expression of ligand-receptor pairs highly expressed in ESCC TME. (**B**) The significantly related ligand–receptor interactions in the ESCC TME inferred by CellChat analysis. Kaplan–Meier curves of overall survival of ESCC microarray cohort stratified by the expression of CCL18 (**C**) and PITPNM3 (**D**). (**E**) A circle plot showing the cell–cell communication network of CCL18-PITPNM3 axis estimated by CellChat. (**F**) CCL18 visualization by immunofluorescence imaging in conjunction with CD68^+^ M1 macrophages or CD206^+^ M2 macrophages

Chemokines regulate the host's response to cancer by recruiting leukocytes or other cells into the TME which may have anti-tumor or tumor-promoting effects [33, 34]. Since biological processes in the TME such as differentiation, inflammation and immune response are mainly mediated by ligand–receptor complexes, we combined ligand–receptor signaling networks [36], signature and differentially expressed genes of each cell type to gain insights into the cross-talks among cell types in the ESCC TME. We focused this study on cytokine- and chemokine-mediated interactions between the tumor cells and the TME. This analysis found the expression of 7 differentially upregulated ligands including T cell marker genes CCL5 and GZMA, monocyte marker genes CCL3, CCL3L1 and CCL4, macrophage marker genes CCL18, GZMA and DC marker gene GZMB, which may impact the infiltration and distribution of the cells in ESCC TME (Fig. 4A left panel). Then, we investigated the expression level of their receptors in the ESCC scRNA-seq dataset and their prognostic significance in the ESCC microarray cohort. CCR1 was highly expressed by monocytes and macrophages, CCR5 was highly expressed by T cells and monocytes, while PITPNM3 was highly expressed by epithelial/tumor cells. (Fig. 4A right panel, and Fig. S1A). We also found ligand receptor pairs CCL3/CCR1, CCL3/CCR5 and CCL18/PITPNM3 have showed significant prognostic value (Log-rank test, *P* < 0.05) (Fig. S1B and C). Analysis of ligand-receptor interactions further identified source and target cell groups with substantial interactions, revealing that CCL3/CCR1/CCR5 and CCL18/PITPNM3 were the most prominent signaling pathways in monocyte and macrophage recruitment. (Fig. 4B).

Among the top upregulated chemokines, CCL18 was highly expressed in tumor and correlated with poor prognosis (Fig. 4C). As a receptor for CCL18, PITPNM3 highly expressed on epithelial and tumor cells in scRNA-seq data (Fig. 4A right panel) and indicated poor prognosis in microarray cohort (Fig. 4D). CCL18-PITPNM3 signaling network inferred from scRNA-seq dataset showed the strongest interaction occurred between epithelial/tumor cells and macrophages (Fig. 4E). CCL18 was also positively correlated with M1 and M2-like TAMs. By using immunofluorescence staining, we confirmed that CCL18 was more frequently expressed by CD68^+^ macrophages (mainly M1-like macrophages) than CD206^+^ macrophages (mainly M2-like macrophages) (Fig. 4F). Subsequently, we analyzed the correlations of CCL18 expression levels with selected clinicopathological features of ESCC patients (Table 1) and found that CCL18 was significantly correlated with tumor stage (*P* = 0.0001). Therefore, these results suggested that CCL18 secreted by TAM might promote the tumor proliferation and lead to poor prognosis in ESCC patients.

### Identification of CCL18 as a potential therapeutic target of ESCC

**Fig. 5:**
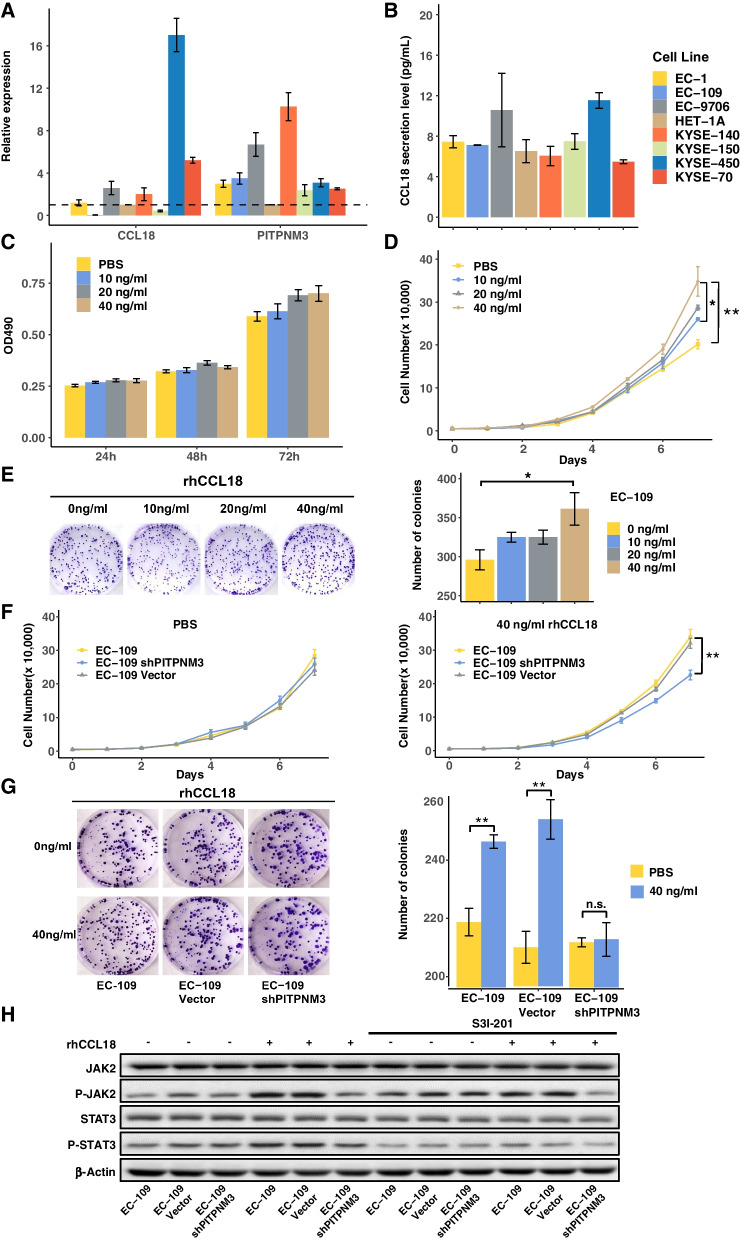
CCL18 promotes proliferation of esophageal squamous carcinoma cell lines. (**A** and **B**) The mRNA expression level of CCL18 and PITPNM3 (**A**), and the secretion level of CCL18 (**B**) in HET-1A and other seven esophageal carcinoma cell lines. (**C** and **D**) The cell viability and growth curve of EC-109 cells in the presence or absence of rhCCL18 at increasing concentrations (10–40 ng/mL), as determined by MTT assay (**C**) or the cell number counting (**D**). (**E**) The colony formation of EC-109 cells in the presence or absence of rhCCL18 at increasing concentrations (10–40 ng/mL). (**F** and **G**) The cell growth curve and clonal formation of EC-109, EC-109 Vector and EC-109 shPITPNM3 cells in the presence or absence of 40 ng/mL rhCCL18. ***P < 0.001, **P < 0.01, *P < 0.05. (**H**) EC-109, EC-109 Vector and EC-109 shPITPNM3 cells were treated with or without 40 ng/mL rCCL18 in the presence or absence of STAT3 inhibitor S3I-201. The expression or phosphorylation of JAK2 and STAT3 were determined by western blot. β-Actin was used as a loading control

As the key functional receptor for CCL18, PITPNM3 is expressed primarily on the cell surface of tumor cells and it has been reported that TAM secreted CCL18 interacted with its receptor PITPNM3 to promote breast cancer metastasis and angiogenesis [16, 17]. To validate the effects of CCL18 on the proliferation of esophageal cancer cells, the expressions of CCL18 and PITPNM3 were detected in various cell lines including the normal esophageal epithelial cell line HET-1A. As shown in Fig. 5A and B, the esophageal cancer cell line EC-109, which expressed PITPNM3 but not CCL18, was selected in the subsequent in vitro experiment. As shown in Fig. 5C and D, rhCCL18 could promote the proliferation of EC-109 cells by MTT assay and the cell number counting. The cell clone formation experiment showed similar results (Fig. 5E). Consistent with the results from the clinicopathological analysis, CCL18 could promote the esophageal cancer cell proliferation. To further investigate the relationship between CCL18 proliferation-promoting effects and its receptor PITPNM3, EC-109 shPITPNM3 cell line with PITPNM3 knockdown was constructed, and the intrinsic function of PITPNM3 on the EC-109 cells were tested. Firstly, we found no difference in cell cycles between EC-109, EC-109 Vector and EC-109 shPITPNM3 cells. Addition of 40 ng/mL rhCCL18 only slightly affected the cell cycle of EC-109 cells (Fig. S5A). Besides, there were no significant differences in apoptosis between EC-109, EC-109 Vector and EC-109 shPITPNM3 cells with or without the treatment of rhCCL18 (Fig. S5B).

The effects of PITPNM3 knockdown on the proliferation and colony formation of EC-109 cells were also tested. There were no significant differences in the cell proliferation detected by cell number counting, MTT assay, and colony formation (Fig. 5G and Fig. S6A and B). Addition of 40 ng/mL rhCCL18 could promote the proliferation and colony formation of EC-109 and EC-109 Vector cells, but not EC-109 shPITPNM3 cells (Fig. 5F and G). These results suggested that CCL18 could promote the proliferation of esophageal cancer cells via its receptor PITPNM3. Besides, the effects of PITPNM3 knockdown on the migration of EC-109 cells were also tested. The knockdown of PITPNM3 did not affect the migration of EC-109 cells by Transwell assay and wound healing assay (Fig. S6C and D).

As reported, CCL18 could promote the migration, invasion, EMT and metastasis of the breast cancer and hepatocellular carcinoma cells through the phosphorylation of PyK2 or NF-κB signaling pathway [16, 18]. Besides, CCL18 could facilitate the proliferation of oral cancer cells through the JAK2/STAT3 signaling pathway [37]. In ESCC, CCL18 could enhance the proliferation and colony formation of EC-109 cells (Fig. 5C, D and E), while exhibit no influence on the migration of EC-109 cells (Fig. S7A and B). Thus, we verified the influence of CCL18 on EC-109 cells through the JAK2/STAT3 signaling pathway. The cell lines EC-109, EC-109 Vector, EC-109 shPITPNM3 and the STAT3 inhibitor S3I-201 were used. There is no significant difference in the expression and phosphorylation of both JAK2 and STAT3 in the three cell lines, while with the addition of rCCL18 (40 ng/mL), the phosphorylation of both JAK2 and STAT3 in EC-109 and EC-109 Vector cells was significantly enhanced, but not in EC-109 shPITPNM3 cells. Thus, CCL18 could activates the JAK2/STAT3 signaling pathway, and the interference of PITPNM3 expression could reduce the phosphorylation level. Further, the enhanced phosphorylation level of JAK2 and STAT3 mediated by rhCCL18 could be reversed by the STAT3 inhibitor S3I-201 (Fig. 5H). Therefore, CCL18/PITPNM3 might promote the proliferation of EC-109 cells via JAK2/STAT3 signaling pathway.

### Design and identification of a CCL18 peptide inhibitor

**Fig. 6:**
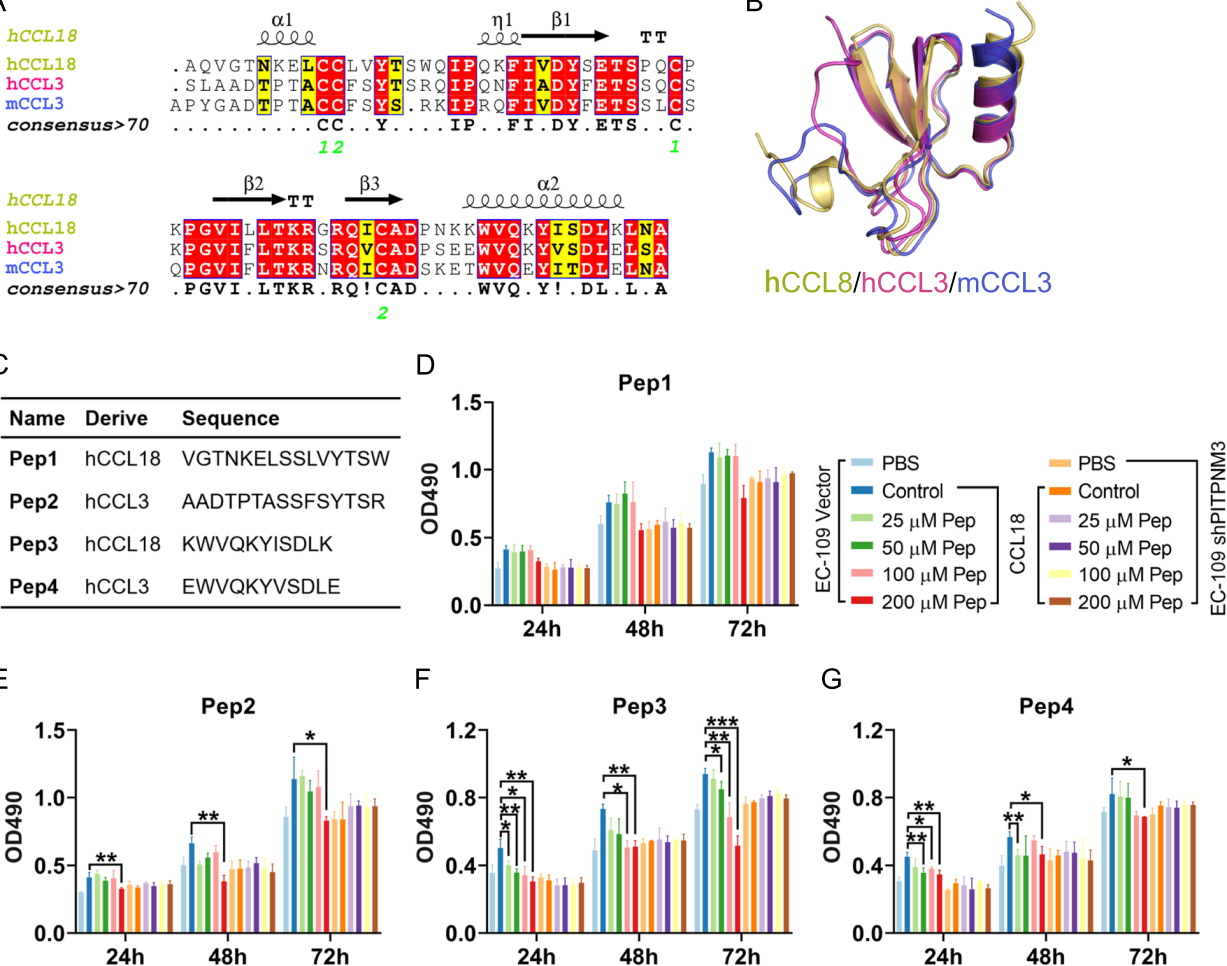
Identification of a CCL18 peptide inhibitor. (**A**) Sequence alignment of human CCL18, human and mouse CCL3 by the online tool ESPript3. The protein structure was labeled according to CCL18. The consensus sequences were listed. The numbers in green represent the cysteine residues to form the disulfide bonds. (**B**) The structure of human CCL18, human and mouse CCL3 were superposed as the sequence alignment using the software MOE. (**C**) The information of peptides derived from human CCL18 or CCL3 selected based on the structure. The effects on the proliferation of EC-109 cells transfected with the vector or shPITPNM3 with or without the existence of 20 ng/mL rhCCL18 by Pep1 (**D**), Pep2 (**E**), Pep3 (**F**) and Pep4 (**G**) in indicated concentrations using the MTT assay. *P < 0.05, **P < 0.01, ***P < 0.001

There is no corresponding gene to CCL18 in mice. After examining all chemokines with sequences and structures similar to CCL18, we discovered the sequence similarity between CCL18 and human CCL3 was 62.3%, and that was 56.52% with mouse CCL3 (Fig. 6A). Further, the structures of the proteins were prepared by the software MOE, and structure superposition was performed. We hypothesized that CCL3 would have a comparable effect to CCL18 in mice, because human CCL18, human and mouse CCL3 exhibited highly similar sequence and structure (Fig. 6B).

We firstly reanalyzed a scRNA-seq data of mouse ESCC model induced by 4-NQO (CRA002118) to test our hypothesis. A total of 19,829 cells from 3 normal mice and 6 ESCC mice (including 3 CIS mice and 3 ICA mice) (Fig. S3A) were selected to compare cell–cell communication patterns between normal and ESCC tumors. In line with our findings, with the human ESCC microarray cohort, cell interactions between epithelial, fibroblasts and myeloid cells significantly increased in the ESCC mice (Fig. S3B-D). The strong autocrine signal of myeloid cells was further highlighted by the cell–cell communication network of the CCL pathway (Fig. S3E), and CCL3 also played a crucial role in myeloid cells recruitment and NK activation (Fig. S3F). We then further verified the expression of mCCL3 in the tissues and macrophages using ESCC mouse model induced by 4-NQO. By using qRT-PCR to measure the expression of CCL3, we found that mCCL3 was overexpressed in ESCC tissues compared to normal esophageal tissues (Fig. S4A). Additionally, ESCC tissues showed upregulation of the CCL3 receptors, CCR1 and CCR5. The immunostaining of the ESCC tissues demonstrated that CCL3 was expressed, and co-localized with the F4/80-positive macrophages (Fig. S4B). The bone marrow-derived macrophages (BMDM) were polarized into M1 or M2 macrophages to evaluate the expression of CCL3. The results demonstrated that BMDM had high expression of CCL3 in mRNA and protein levels, and M1 macrophages secreted more CCL3 than that from M2 macrophages (Fig. S4C).

We further developed a CCL18 inhibitor to confirm the contribution of macrophages and CCL18 to the development of ESCC. The potential functional fragments from both human CCL18 and CCL3 were selected based on the sequence alignment and structures, since the CCL18 inhibitor may also be effective in the mouse model (Fig. 6C). Pep 1–4 from the N and C terminal of CCL18 and CCL3 were selected and synthesized. We initially investigated the inhibitory effects of the peptides on the proliferation of EC-109 cells by MTT, as rhCCL18 could facilitate the proliferation of the ESCC cells dependent on its interaction with the receptor PITPNM3. Similar as rhCCL18, the peptides also showed no obvious effects on the EC-109 shPITPNM3 cells, suggesting the peptides themselves had no effects on cell proliferation. However, Pep3 could significantly reverse the promotion of EC-109 cell proliferation by rhCCL18 in a dose-dependent manner (Fig. 6F). Pep4, which had similar structure and sequence to Pep3, had less potent effects (Fig. 6G), whilst the effects of Pep1 and Pep2 were only effective at a high concentration of 200 μM (Fig. 6D and E). Thus, Pep3 was selected as a CCL18 inhibitor and further explored in the ESCC mouse model.

### The peptide inhibitor Pep3 significantly inhibit the tumor growth in the spontaneous ESCC mouse model

**Fig. 7:**
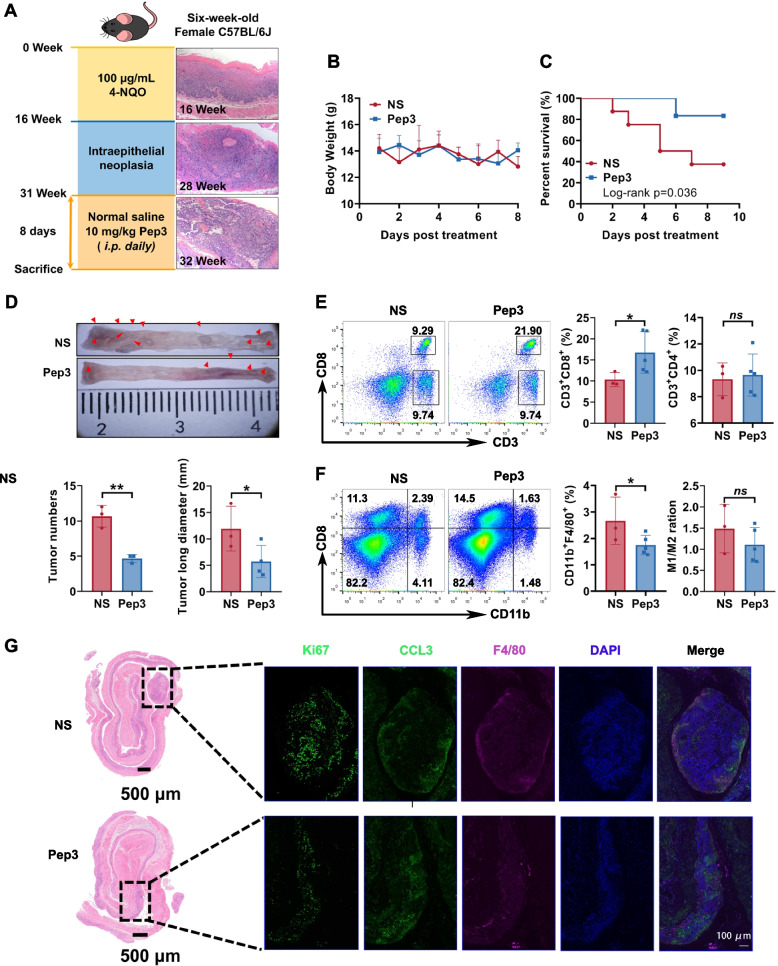
Tumor inhibition of the 4-NQO induced ESCC mouse model by a CCL18 peptide inhibitor. (**A**) Schematic illustration of the establishment and verification of the ESCC model. The ESCC mice were randomly divided into the NS or Pep3 treatment groups, and treated with 10 mg/kg of Pep3 for 8 days. (**B**) The body weight of the ESCC mice treated with NS or Pep3. (**C**) Survival curve of the ESCC mice (*n* = 8 for NS, *n* = 7 for Pep3). (**D**) Tumors of represented with the whole esophageal tissues, the numbers of the tumors and the total long diameter of the tumors were recorded (*n* = 3–4). *P < 0.05, **P < 0.01. (**E**) The representative flow cytometry plots and summary data of CD8^+^ or CD4^+^ T cells in the spleen of the ESCC mice (*n* = 3 or 5). ns, no significance, *P < 0.05. (**F**) The representative flow cytometry plots and summary data of macrophages or M1/M2 ratio in the spleen of the ESCC mice (*n* = 3 or 5). ns, no significance, *P < 0.05. (**G**) The H&E staining and immunostaining of the esophageal tissues of ESCC mice after treatment. The representative figures were obtained from serial section slides

The putative spontaneous ESCC mouse model was established, and intraepithelial neoplasia developed in the esophageal tissues at the 24^th^ week, which was confirmed by H&E staining (Fig. 7A). The mice that developed ESCC spontaneously were separated into two groups randomly and treated with 10 mg/kg of Pep3 or vehicle.

The administration of Pep3 demonstrated no obvious effects on the body weight of the ESCC mice (Fig. 7B), while it could significantly prolong the survival of the mice (*P* = 0.036) (Fig. 7C). The treatment of Pep3 significantly restrained the tumor progression, resulting in a considerable decrease in both the overall length diameter and the number of tumors (Fig. 7D). Compared with the control group, mice treated with Pep3 had a higher percentage of CD8^+^ T cells in the splenocytes, suggesting the treatment exert potent and effective immune response. The treatment had no effects on the percentage of CD4^+^ T cells (Fig. 7E). In the meantime, we analyzed the macrophages in the spleen. Pep3 reduced the overall number of macrophages but had no effect on the ratio of M1/M2 macrophages (Fig. 7F). Additionally, H&E staining and immunostaining were used to detect cell proliferation and immune cell infiltration in the esophageal tissues. The Pep3 treatment significantly restricted tumor development in the esophageal tissues (Fig. 7G), which was consistent with the tumor inhibition shown in Fig. 7D. In contrast, the control group had an elevated expression of Ki67. Corresponding to the elevated expression of CCL18 and macrophage infiltration in the human ESCC tissues, Pep3 significantly inhibited CCL3 secretion and macrophage infiltration in ESCC tissues of the mice. Besides, Pep3 could significantly inhibit the expression of PITPNM3 at both the mRNA and protein levels in the ESCC tissues of 4-NQO-induced mice (Fig. S8). Above all, CCL18/CCL3 blockade could elicit significant antitumor effects in ESCC through preventing the infiltration of tumor-associated macrophages and proliferation of ESCC cancer cells, which might be mediated by CCL3/CCR1, CCL3/CCR5 and CCL18/PITPNM3 pathways.

## Discussion

So far, the development of early diagnostic biomarkers and therapeutic targets for ESCC has been relatively slow, which has hampered clinical outcome. The TME, which reflects the immune response in a number of malignancies and plays a significant role in the genesis and progression of cancer [12, 38, 39]. Our research provides a comprehensive understanding of the cellular compositions, cell–cell interactions, and molecular mediators underlying ESCC carcinogenesis. The resulting computational predictions and experimental findings not only substantially recapitulate known biology, but also provide proof-of-principle examples for the integration of scRNA-seq data and microarray datasets as a useful resource for future therapeutic targets identification and development in pharmaceutical fields.

To characterize the whole TME landscape of ESCC, we first identified that the differentially expressed genes in ESCC revealed the crucial function of ECM-related genes (such as MMPs, cytokines, and chemokines) and immune checkpoints during tumor invasion and EMT (Fig. 1B-D). Previous findings revealed a positive contribution of IL-17 to the modulation of neutrophil-mediated antitumor immunity in ESCC [40], and several MMPs were known as downstream of STAT3 and NF-κB signaling pathway and may promote the proliferation and invasion of ESCC [41]. In accordance with a previous study, the PI3K/Akt/mTOR pathway was also upregulated in ESCC [42]. As the main component of exaction extracellular matrix (ECM), the collagen genes are often crosslinked and linearized leading to increased stiffening of the tissue during tumor progression [43]. These genes were reported to characterize immune negative feedback-loop, which could lead to immunosuppression or even immune exclusion in TME [44, 45]. These findings were further validated by cell-type specific gene signatures derived from ESCC scRNA-seq dataset (Fig. 2B).

Our integrated analysis of single cell RNA-seq and microarray data not only defined the ESCC TME, but also provided novel insights into the cell–cell interactions between tumor and immune cells in ESCC [46]. Compared with adjacent peri-tumor sites, enrichment of fibroblasts, macrophages, monocytes, and endothelial cells at ESCC tumor sites indicated chronic inflammation, which created a fertile niche for carcinogenesis (Fig. 2C). Mapping cell interactions across tumor and peritumor sites provides valuable information to unveil the multi-faceted role of the immune system in ESCCs, and its involvement in tumor escape mechanisms [47] which may be overlooked by previous studies focusing on only one or several types of TME cells [48, 49]. In ESCC tumors, the increased cell interactions occurred mostly within and between the epithelial, fibroblasts and immune cells. Moreover, differential cell interaction analysis identified signaling from macrophages, DCs, and monocytes to epithelial/tumor and T cells was strengthened in ESCC tumors (Fig. 3D).

Combing clinical information in the ESCC microarray cohort, we were able to uncover prognostically relevant cell subtypes and cell communication ligands by dissecting the ESCC TME. In multiple solid tumors, TAMs are actively recruited to the TME through paracrine communication and chemotaxis with the tumor cells [16, 19, 36]. CCL18 is mainly secreted by antigen-presenting cells of the innate immune system, such as DCs, monocytes, and macrophages. It can act on the adaptive immune cells, and recruit naïve T cells, Tregs, or even B cells [50, 51]. TAMs were thought to closely resemble the M2 phenotype; however, recent findings suggested that this binary polarization model was oversimplified and a spectrum model of TAM phenotypes has been proposed instead [52]. TAM is highly plastic within TME, where they display different characteristics and functions and has mixed expression profiles ranging from M1 to M2 [53].

Comparative analysis of cell communication further screened prognostic ligand receptor pairs regulating ESCC TME including CCL3/CCR1, CCL3/CCR5 and CCL18/PITPNM3 (Fig. S1). We finally confirmed CCL18-PITPNM3 signaling network showed strongest interaction occurred between epithelial/tumor cells and macrophages based on cell interaction network inferred from ESCC scRNA-seq data (Fig. 4E). Chemokines play important roles during the cell migration between different organs and tissues. Therefore, it would be helpful to develop potential cancer treatments involving the immunostimulatory action of cytokines in order to address cytokine imbalance. They can regulate host response to cancer by directing leukocytes or other cells into the TME to elicit anti-tumor or tumor-promoting effects [33, 34]. The role of CCL18 in cancer progression is controversial, it was reported that CCL18 could directly promote invasion, metastasis and angiogenesis in breast cancer, pancreatic cancer and ovarian cancer [16, 17], but CCL18 was associated with prolonged survival in patients with gastric cancer [54]. Here we found that CCL18 was elevated in tumor tissues of ESCC, negatively correlated with the patient survival, and positively correlated with tumor developing stage (Table 1), which corresponded with its high expression level on TAMs infiltration (Fig. 2B). As a receptor of CCL18, PITPNM3 mainly expressed in human retina, brain, spleen, and breast cancer cells [55]. It was reported that CCL18/PITPNM3 interaction could also promote liver cancer migration, invasion, EMT, and the progression of pancreatic ductal carcinoma [56]. Similarly, we discovered that CCL18 could promote proliferation of esophageal cancer cells via its interaction with PITPNM3. Although we discovered the CCL18 was mainly expressed by M1 macrophages, the role of M2 macrophages shall not be fully ruled out. It was uncovered that CCL18 caused the maturation of cultured monocytes to macrophages in the M2 spectrum [57]. Therefore, CCL18 secreted by macrophages in the tumor tissues of ESCC could promote the proliferation of esophageal cancer cells and led the polarization of macrophages toward M2 phenotype.

Considering the high similarities of structures and sequences of CCL3 and CCL18, we designed and evaluated blocking peptides based on the functional fragments of both chemokines. The Pep3 constructed from the N terminus of hCCL18 was selected due to its greatest inhibition effect on EC-109 cell proliferation (Fig. 6). The antitumor efficacy of CCL18 blocking peptide Pep3 was further validated in vitro and in vivo, using a spontaneous ESCC mice model induced by 4-NQO. Pep3 is, to our knowledge, the first peptide to inhibit CCL18 and greatly reduce the ESCC cancer progression. This peptide may reduce esophageal carcinogenesis by inhibiting TAM infiltration and recruitment via CCL18/PITPNM3 or CCL3/CCR1/CCR5 signaling, hence enhancing the CD8^+^ T cell response (Fig. 7).

Furthermore, the pan-cancer analyses between CCL18 and immune checkpoints expression have important implications for cancer immunotherapy. As a number of inhibitory immunoreceptors have been identified and studied, 40 known immune checkpoint genes were collected, and were correlated with the expression of CCL18 (Fig. S9). We found 26 checkpoints were highly co-expressed with CCL18 including but not limited to PD-L1 (CD274), LAG3, TIGIT and CTLA4. Compared with other 33 cancer types in The Cancer Genome Atlas (TCGA) (Fig. S10), CCL18 was co-expressed with multiple immune checkpoints in 14 cancers. This demonstrated the great potential of CCL18 to be served as a pan-cancer target for future immunotherapy.

There are major efforts to develop therapeutic strategies to overcome immune resistance by using a combination of checkpoint blockers, and to improve drug efficacies and response rate of patients. Recent reports have indicated the strategies to improve phagocytic ability and reduce tumor growth via blocking PD-1 on TAMs [58] or promoting M2 macrophage polarization [59, 60]. TME changes not only influence tumor progression, but also dramatically influence the efficacy of cancer therapy. Monocyte derived macrophages build an essential inflammatory niche shaping tumor immune microenvironment. Recent progress defined the molecular landscapes and mechanisms of macrophage differentiation [61] held the promise to uncover its heterogeneity and functional roles within tumors. TAMs secrete cytokines and chemokines that can suppress T cell recruitment and activation, thereby promoting resistance to immune checkpoint inhibition. Novel therapeutic techniques, as demonstrated here, have the potential to synergize with checkpoint inhibitors, chemotherapy, and/or radiation therapy to enhance the overall efficacy of cancer treatment.

## Conclusion

**Fig. 8:**
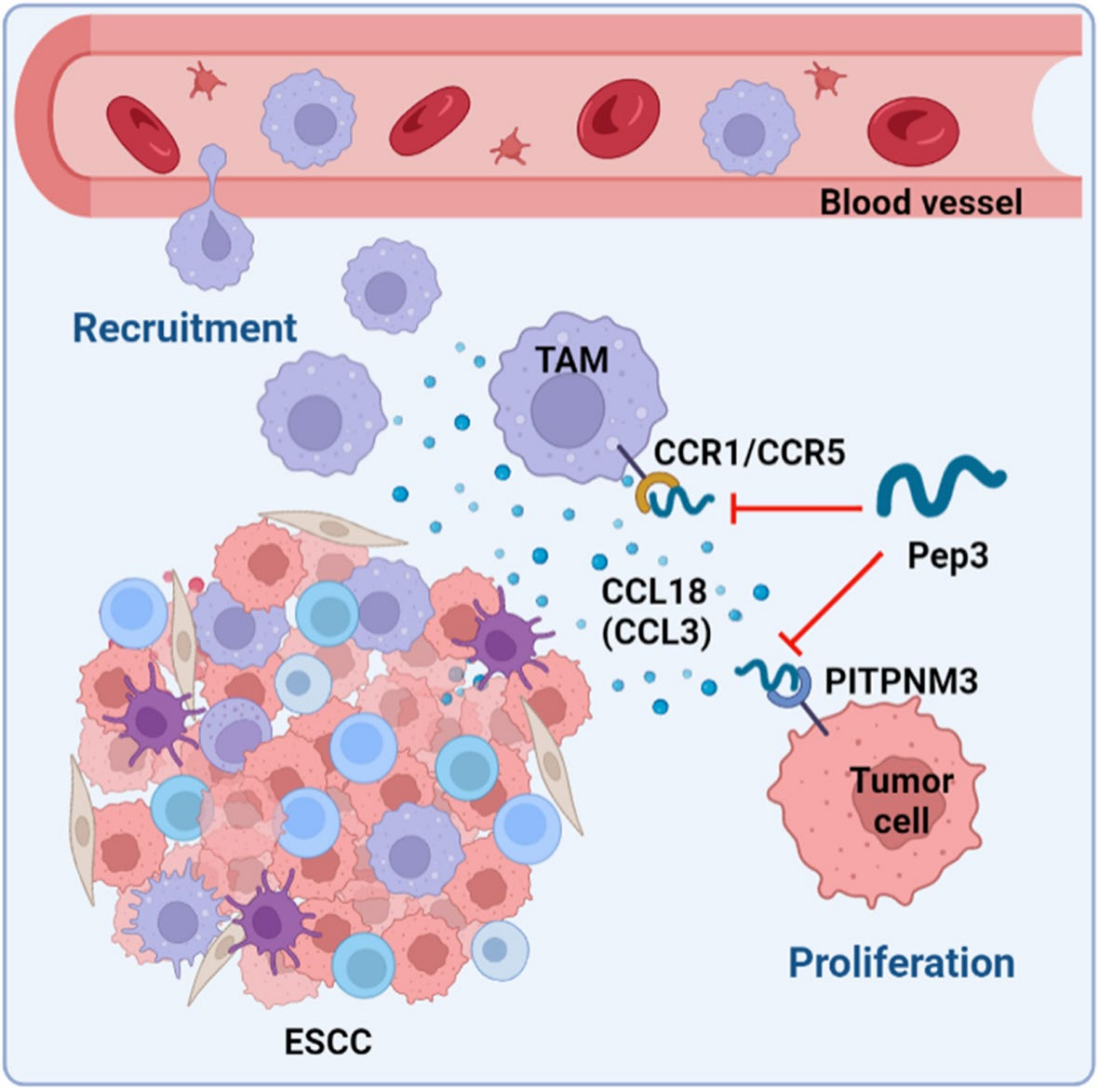
Schematic illustration of the possible antitumor mechanism of CCL18/CCL3 blockade. CCL18 and CCL3 in the ESCC TME are secreted by TAMs and other myeloid cells. Pep3 may elicit antitumor effects in ESCC by preventing infiltration of tumor-associated macrophages (TAM) and proliferation of ESCC cancer cells, via CCL3/CCR1/CCR5 and CCL18/PITPNM3 pathways

In summary, our study provided a comprehensive landscape of TME in ESCC by characterizing the cell–cell interactions dynamics of ESCC TME, thus provided novel insights to understand the co-evolution of tumor and immune system in ESCC. We identified that CCL18, mainly secreted by macrophages, and may serve as a target for the diagnosis and immunotherapy of ESCC by combining bioinformatic analysis and in vitro validation of the associated signaling pathway. We designed and screened a CCL18 blockade peptide and validated its antitumor activity in vitro and in vivo. As summarized in Fig. 8, CCL18 blockade might inhibit esophageal carcinogenesis by preventing TAMs recruitment and cancer cell proliferation.

## Supplementary Information

**Additional file 1:** **FigureS1.** Expression,distribution, and prognostic significance of CCL3, CCL18 and their receptors inESCC TME. (A) Bubble heatmap showing expression levels of CCL3,CCL18 and their receptors in different cell types and locations based on ESCC scRNA-seqdataset. Dot size indicates percentage of expressing cells, colored based onnormalized expression levels. (B) Kaplan-Meier curves of overall survival ofESCC microarray cohort stratified by the expression of CCL3 and its receptorsCCR1 and CCR5. (C) Kaplan-Meier curves of overall survival of ESCC microarraycohort stratified by the expression of CCL18 receptors ACKR1, ERG28 and CCR8. **FigureS2.** Cell-cell communication network of ESCC. Differential number of possible interactions and interaction strength(edge weight) between the five major cell types between peri- and tumor sites(A and B). Number of possible interactions between the 11 cell types in peri-and tumor sites (C and D). **Figure S3.** Cell-cellcommunication in 4-nitroquinoline 1-oxide (4NQO) inducedmouse ESCC model. (A) UMAP visualization of 19,829 cells from 3 normal mice, 3 carcinoma *in situ*(CIS) mice and 3 invasive carcinoma (ICA) mice (CRA002118). (B) Differential numberof possible interactions between any two cell populations. Red (positivevalues) and blue (negative values) in the color bar indicate higher number ofpredicted interactions in normal and tumor mice samples, respectively. (C)A circle plotshowing the number of possible interactions in normal mice estimated byCellChat. (D) A circle plot showing the number of possible interactions in CIS and ICAmice estimated by CellChat. (E)A circle plot showing the cell-cell communicationnetwork of CCL pathway estimated by CellChat. (F)The significantly related ligand–receptor interactions of CCL pathway in theESCC mouse model inferred by CellChat analysis. **Figure S4.** The expression of CCL3 or its receptor inthe ESCC tissues or macrophages. (A)The mRNA expression level of CCL3, CCR1 and CCR5 in the ESCC tissues of 4-NQOinduced mice with ESCC by qPCR. (B) The expression of CCL3 and co-localizationwith macrophages (F4/80) in the mouse ESCC tissues. Representative views were shown. (C) The expression of CCL3 in BMDM polarized M1 or M2 macrophages inmRNA and protein levels. (*P < 0.05, ***P < 0.001). **Figure S5.**The cell cycle and apoptosis of EC-109, EC-109 Vector and EC-109 shPITPNM3cells. (A) Cell cycles of EC-109, EC-109 Vectorand EC-109 shPITPNM3 cells treated with or without 40ng/mL CCL18 for 72 h. *P< 0.05. (B) Flow cytometry analysis of the apoptosis of EC-109, EC-109Vector and EC-109 shPITPNM3 cells treated with or without 40ng/mL CCL18 for 72h. n.s., no significance. **Figure S6.** The proliferation, colony formation and migration analysis ofEC-109, EC-109 Vector and EC-109 shPITPNM3 cells. (A) The cell growth curve and cell viability of EC-109, EC-109 Vector andEC-109 shPITPNM3 cells determined by the cell number counting and MTT assay. (B)The colony formation of EC-109, EC-109 Vector and EC-109 shPITPNM3 cells toform clones. (C) Representative results of the Transwell assay of EC-109,EC-109 Vector and EC-109 shPITPNM3 cells at 48 h. Bar = 200 μm. (D) Representativeimages of the wound-healing assay of EC-109, EC-109 Vector and EC-109 shPITPNM3cells after 48 h. n.s., no significance. **Figure S7.** (A)Representative results of the Transwell assay of EC-109 cells in the presenceor absence of rhCCL18 at increasing concentrations (10–40 ng/mL) for 48 h. (B)Representative images of the wound-healing assay of EC-109 cells in thepresence or absence of rhCCL18 at increasing concentrations (10–40 ng/mL) at 0h and 48 h. n.s., no significance. **Figure S8.** The expression of PITPNM3 inthe ESCC tissues with or without Pep3 treatment. (A) The mRNA expression level of PITPNM3 in the ESCC tissues of 4-NQOinduced mouse model by qPCR. (B) Representative images of PITPNM3 staining inthe ESCC tissues, bar = 50 μm. ***P < 0.001. **Figure S9.** Correlationof CCL18 with immune scores, CD8 T cells and 40 known immune checkpoints in ESCC. The correlationmap between 40 common immune checkpoint genes andthe expression of CCL18 is as follows: *** *P* < 0.001, ** *P*< 0.01, * *P* < 0.05. Most correlated immune checkpoint genes arehighlighted in red. **Figure S10.** Correlation of CCL18 with known immunecheckpoints. Calculated from RNA-seqexpression data from The Cancer Genome Atlas (TCGA)datasets across 33 different cancer types. *** *P* < 0.001, ** *P*< 0.01, * *P* < 0.05.

## Data Availability

The dataset and associated source codes supporting the conclusions of this article is available from the corresponding author upon request.

## Abbreviations

ESCC: Esophageal squamous cell carcinoma
TME: Tumor microenvironment
EMT: Epithelial mesenchymal transformation
CCL18: Chemokine C–C motif ligand 18
IL-17: Interleukin-17
TCGA: The Cancer Genome Atlas
TAMs: Tumor associated macrophages
DCs: Dendritic cells
FACS: Fluorescence-activated cell sorting
IHC: Immunohistochemistry
eBayes: Empirical Bayes
DEGs: Differentially expressed genes
UMAP: Uniform manifold approximation and projection
FBS: Fetal bovine serum
PITPNM3: PITPNM Family Member 3
