# Supplementary material for: Integrative analysis of bulk and single-cell gene expression profiles to identify tumor-associated macrophage-derived CCL18 as a therapeutic target of esophageal squamous cell carcinoma
Source: J Exp Clin Cancer Res. 2023 Feb 27;42:51. doi: 10.1186/s13046-023-02612-5 (PMC9969639; doi:10.1186/s13046-023-02612-5)
Supplement: Supplementary file 1 — Additional file 1: FigureS1. Expression,distribution, and prognostic significance of CCL3, CCL18 and their receptors inESCC TME. (A) Bubble heatmap showing expression levels of CCL3,CCL18 and their receptors in different cell types and locations based on ESCC scRNA-seqdataset. Dot size indicates percentage of expressing cells, colored based onnormalized expression levels. (B) Kaplan-Meier curves of overall survival ofESCC microarray cohort stratified by the expression of CCL3 and its receptorsCCR1 and CCR5. (C) Kaplan-Meier curves of overall survival of ESCC microarraycohort stratified by the expression of CCL18 receptors ACKR1, ERG28 and CCR8. FigureS2. Cell-cell communication network of ESCC. Differential number of possible interactions and interaction strength(edge weight) between the five major cell types between peri- and tumor sites(A and B). Number of possible interactions between the 11 cell types in peri-and tumor sites (C and D). Figure S3. Cell-cellcommunication in 4-nitroquinoline 1-oxide (4NQO) inducedmouse ESCC model. (A) UMAP visualization of 19,829 cells from 3 normal mice, 3 carcinoma in situ(CIS) mice and 3 invasive carcinoma (ICA) mice (CRA002118). (B) Differential numberof possible interactions between any two cell populations. Red (positivevalues) and blue (negative values) in the color bar indicate higher number ofpredicted interactions in normal and tumor mice samples, respectively. (C)A circle plotshowing the number of possible interactions in normal mice estimated byCellChat. (D) A circle plot showing the number of possible interactions in CIS and ICAmice estimated by CellChat. (E)A circle plot showing the cell-cell communicationnetwork of CCL pathway estimated by CellChat. (F)The significantly related ligand–receptor interactions of CCL pathway in theESCC mouse model inferred by CellChat analysis. Figure S4. The expression of CCL3 or its receptor inthe ESCC tissues or macrophages. (A)The mRNA expression level of CCL3, CCR1 and CCR5 in the E [file 13046_2023_2612_MOESM1_ESM.docx]

**SUPPLEMENTARY MATERIAL**

**Figure S1.** **Expression, distribution, and prognostic significance of CCL3, CCL18 and their receptors in ESCC TME.** (A) Bubble heatmap showing expression levels of CCL3, CCL18 and their receptors in different cell types and locations based on ESCC scRNA-seq dataset. Dot size indicates percentage of expressing cells, colored based on normalized expression levels. (B) Kaplan-Meier curves of overall survival of ESCC microarray cohort stratified by the expression of CCL3 and its receptors CCR1 and CCR5. (C) Kaplan-Meier curves of overall survival of ESCC microarray cohort stratified by the expression of CCL18 receptors ACKR1, ERG28 and CCR8.

**Figure S2. Cell-cell communication network of ESCC.** Differential number of possible interactions and interaction strength (edge weight) between the five major cell types between peri- and tumor sites (A and B). Number of possible interactions between the 11 cell types in peri- and tumor sites (C and D).

**Figure S3. Cell-cell communication in** **4-nitroquinoline 1-oxide (4NQO) induced mouse ESCC model.** (A) UMAP visualization of 19,829 cells from 3 normal mice, 3 carcinoma *in situ* (CIS) mice and 3 invasive carcinoma (ICA) mice (CRA002118). (B) Differential number of possible interactions between any two cell populations. Red (positive values) and blue (negative values) in the color bar indicate higher number of predicted interactions in normal and tumor mice samples, respectively. (C) A circle plot showing the number of possible interactions in normal mice estimated by CellChat. (D) A circle plot showing the number of possible interactions in CIS and ICA mice estimated by CellChat. (E) A circle plot showing the cell-cell communication network of CCL pathway estimated by CellChat. (F) The significantly related ligand–receptor interactions of CCL pathway in the ESCC mouse model inferred by CellChat analysis.

**Figure S4. The expression of CCL3 or its receptor in the ESCC tissues or macrophages.** (A) The mRNA expression level of CCL3, CCR1 and CCR5 in the ESCC tissues of 4-NQO induced mice with ESCC by qPCR. (B) The expression of CCL3 and co-localization with macrophages (F4/80) in the mouse ESCC tissues. Representative views were shown. (C) The expression of CCL3 in BMDM polarized M1 or M2 macrophages in mRNA and protein levels. (**P* < 0.05, ****P* < 0.001).

**Figure S5. The cell cycle and apoptosis of EC-109, EC-109 Vector and EC-109 shPITPNM3 cells.** (A) Cell cycles of EC-109, EC-109 Vector and EC-109 shPITPNM3 cells treated with or without 40ng/mL CCL18 for 72 h. **P* < 0.05. (B) Flow cytometry analysis of the apoptosis of EC-109, EC-109 Vector and EC-109 shPITPNM3 cells treated with or without 40ng/mL CCL18 for 72 h. n.s., no significance.

**Figure S6.** **The proliferation, colony formation and migration analysis of EC-109, EC-109 Vector and EC-109 shPITPNM3 cells.** (A) The cell growth curve and cell viability of EC-109, EC-109 Vector and EC-109 shPITPNM3 cells determined by the cell number counting and MTT assay. (B) The colony formation of EC-109, EC-109 Vector and EC-109 shPITPNM3 cells to form clones. (C) Representative results of the Transwell assay of EC-109, EC-109 Vector and EC-109 shPITPNM3 cells at 48 h. Bar = 200 μm. (D) Representative images of the wound-healing assay of EC-109, EC-109 Vector and EC-109 shPITPNM3 cells after 48 h. n.s., no significance.

**Figure S7.** (A) Representative results of the Transwell assay of EC-109 cells in the presence or absence of rhCCL18 at increasing concentrations (10–40 ng/mL) for 48 h. (B) Representative images of the wound-healing assay of EC-109 cells in the presence or absence of rhCCL18 at increasing concentrations (10–40 ng/mL) at 0 h and 48 h. n.s., no significance.

**Figure S8. The expression of PITPNM3 in the ESCC tissues with or without Pep3 treatment.** (A) The mRNA expression level of PITPNM3 in the ESCC tissues of 4-NQO induced mouse model by qPCR. (B) Representative images of PITPNM3 staining in the ESCC tissues, bar = 50 μm. ****P* < 0.001.

**Figure S9. Correlation of CCL18 with immune scores, CD8 T cells and 40 known immune checkpoints in ESCC.** The correlation map between 40 common immune checkpoint genes and the expression of CCL18 is as follows: *** *P* < 0.001, ** *P* < 0.01, * *P* < 0.05. Most correlated immune checkpoint genes are highlighted in red.

**Figure S10. Correlation of CCL18 with known immune checkpoints.** Calculated from RNA-seq expression data from The Cancer Genome Atlas (TCGA) datasets across 33 different cancer types. *** *P* < 0.001, ** *P* < 0.01, * *P* < 0.05.
